# Extrathoracic heart in northern Cameroon: a case report

**Published:** 2009-06-10

**Authors:** Tantchou Tchoumi Jacques Cabral, Alessandro Giamberti, Gianfranco Butera, Alessandro Frigiola, Ambassa Jean Claude

**Affiliations:** 1 Istituto policlinico San Donato Via Morandi 30, 29007 San Donato Milanese; 2 Cardiac unit, Shisong St. Elizabeth catholic general hospital P.O Box 8 Kumbo

**Keywords:** ectopia cordis

## Abstract

Sternal clefts, ectopia cordis, and Cantrell’s pentalogy continue to be very rare congenital anomalies in pediatric surgery. The prenatal diagnosis is easily made with ultrasound by visualizing the heart outside the thoracic cavity. Ectopia cordis is frequently associated with other congenital defects involving multiple organ systems. We report a case of ectopia cordis with successful surgical correction on a 7 months old child from northern Cameroon.

## Background

Exthoracic heart (ectopia cordis) is defined as an anomaly in which the fetal heart lies outside the thoracic cavity. It is a rare congenital abnormality with an incidence of 5.5–7.9 per 1 million live births [1]. Ectopia cordis (EC) may occur as an isolated malformation or it may be associated with other ventral body wall defects affecting the thorax, abdomen or both. The cause of EC is currently unknown, and most cases are sporadic [2] Four types of EC have been described according to the position of the heart; anterior to the sternum (thoracic: 65%), between the thorax and abdomen (thoracoabdominal: 20%), within the abdomen (abdominal: 10%) or in the neck (cervical: 5%) [3, 4]. We report a case of EC with a successful surgical correction.

## Patient and case report

**Figure 1:**
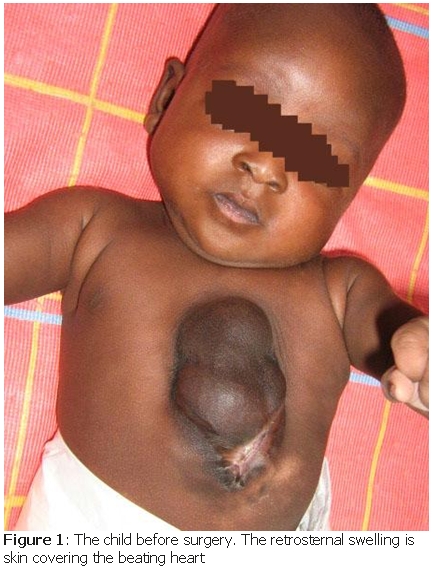
The child before surgery. The retrosternal swelling is skin covering the beating heart

Miss D is a 18 years old lady in her first pregnancy, native from the far northern part of Cameroon, consulting in a health center for the first time at 38 weeks of pregnancy for lower abdominal pain. Few hours later, she delivered a baby boy of normal weight and height. The nurses noticed that the child may have the heart outside the thoracic cavity, covered only by skin. However, as with normal deliveries, the mother and the child were sent home after 4 days. No attention was given to the extraordinary malformation. Seven months later, in September 2008, an English surgeon saw the case during an ambulatory consultation (Figure 1).

**Figure 2:**
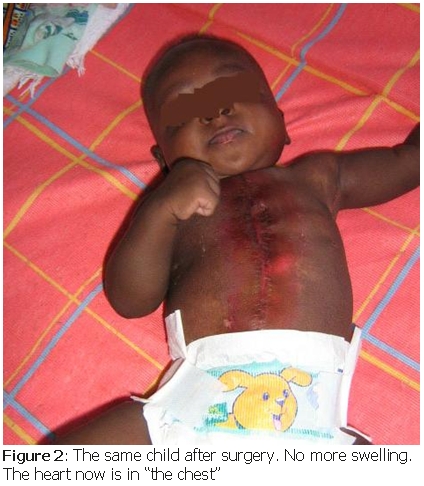
The same child after surgery. No more swelling. The heart now in “the chest”

Required administrative paperwork were diligently conducted and the patient was transferred to the Istituto Polyclinico San Donato in Milan (Italy), a centre specialized in the correction of this malformation. The cardiac Doppler-echocardiography performed as part of the patient initial evaluation showed no other associated pathology. A surgical correction was done, involving a plastic surgeon, a paediatric general surgeon and a paediatric cardiac surgeon. The surgery went well; the child was extubated the 2^nd^ day after surgery and discharged with the heart inside “the chest” (Figure 2). He returned to Cameroon and is expected in Shisong cardiac centre for follow-up.

## Discussion

This is the very first documented case diagnosed in Cameroon and in central Africa. It was very surprising that the child reached 7 months, certainly because the pathology was not associated with any cardiac malformation. Gokhan Yildirim et al [5] reported that EC is frequently associated with other congenital defects involving multiple organ systems. Ventricular septal defects and tetralogy of Fallot are the most common associated intracardiac defects, while omphalocele is the most common associated abdominal wall defect. Most infants are stillborn or die within the first few hours or days of life. It is very important to mention that being very risky, and though to be unsuccessful in the literature the surgical correction was performed with a good outcome [6]. The prognosis of the case being poor, regular follow up will be necessary for the patient in other to detect and correct potential complications. The prenatal diagnosis of EC is easily made with ultrasound, which allows visualization of the heart outside the thoracic cavity [7]. The procedure unfortunately can be performed only in very few Cameroonian hospitals because of the lack of specialists, equipment and poverty. The pathogenesis of EC and coexisting anomalies has been the subject of research, and there are many theories that attempt to explain this anomaly, including the amniotic band theory, the vascular disruption theory, the theory of a defect in the foetal folding process and the theory of disturbances of field development [8]. Developmental fields are those units of the embryo in which the development of a particular complex structure is determined and controlled in a coordinated, temporally synchronous and hierarchical manner [9].

## Conclusion

Ectopia cordis is a rare congenital pathology that can be found everywhere. The progress in paediatric cardiac surgery is changing the prognosis of this malformation. Prenatal ultrasound is still the best diagnostic tool for the pathology. This case also highlights the need to strengthen the existing referral mechanisms and raise the awareness of the health personnel on the prompt referral of unusual health issues.

## Conflict of interest

No conflict of interest declared.

## Acknowledgement

I wanted to use the opportunity to thank Cuore Fratello, Associazione Bambini Cardiopatici nel mondo, the surgical teams of San Donato Policlinico, Dr Abiguail, the Tertiary sisters of St. Francis for their assistance in the management of the case and their assistance in the conception of the manuscript.
